# Image Segmentation Techniques for Healthcare Systems

**DOI:** 10.1155/2019/2723419

**Published:** 2019-04-02

**Authors:** Orazio Gambino, Vincenzo Conti, Sergio Galdino, Cesare Fabio Valenti, Wellington Pinheiro dos Santos

**Affiliations:** ^1^Dept. of Engineering, University of Palermo, Palermo, Italy; ^2^Faculty of Engineering and Architecture, University of Enna KORE, Enna, Italy; ^3^Polytechnique School of Pernambuco, University of Pernambuco, Recife, Brazil; ^4^Dept. of Mathematics and Informatics, University of Palermo, Palermo, Italy; ^5^Dept. of Biomedical Engineering, Federal University of Pernambuco, Recife, Brazil

The present special issue of the *Journal of Healthcare Engineering* collects articles written by researchers scattered around the world who belong to the academic and industrial environments. The papers of this special issue have been selected by a rigorous peer-reviewing process with the support of at least two reviewers per paper, along with the opinion written in the final decision by a component of the editorial staff. Different methods on biomedical image segmentation dedicated to healthcare systems have been developed regarding, for example, the fields of machine learning, deformable models, fuzzy models, and so on. Such methods have been applied on different biomedical image modalities (MRI, CT, mammograms, optical coherence tomography, and others) of various anatomical districts, such as the brain, thyroid, lung, and breast. J. Gauci et al. present an automatic approach to determine the temperature of the body's extremities by means of thermal images in diabetic patients. The approach is based on morphological operations, and geometric transformations are aimed at automatically extracting the required data from 44 predefined regions of interest. The method is tested on data from 395 participants. A correct extraction in around 90% of the images was achieved. A. Bougacha et al. investigate a novel classification method for 3D multimodal MRI glioblastoma tumor characterization. They propose the segmentation problem as a linear mixture model (LMM). First, they provide a nonnegative matrix *M* from every MRI slice in every segmentation process' step (matrix used as an input for the first segmentation process to extract the edema region from T2 and FLAIR modalities); after that, they extract edema's region from T1c modality, generate the matrix *M*, and segment the necrosis, the enhanced tumor, and the nonenhanced tumor regions. In the segmentation process, they apply a rank-two NMF clustering. C. Wang et al. quantify the subregional alveolar bone changes during orthodontic tooth movement with a novel method. Orthodontic tooth movement (OTM) is the result of the region-specific bone modeling under a load. Quantification of this change in the alveolar bone around a tooth is a basic requirement to understand the mechanism of orthodontics. They have used 12 Sprague-Dawley (SD) rats as an orthodontic model, and one side of the first upper molar has been used to simulate OTM. The alveolar bone around the mesial root has been reconstructed from in vivo micro-CT images and separated from other parts of the alveolar bone with two semicylinder filters. The amount and rate of OTM, bone mineral density (BMD), and bone volume (BV) around the root have been calculated and compared at 5 time points. C. L. Toledo Peral et al. present an application for skin macules characterization and could be the background of a future diagnosis-assistance-tool for educational and preventive assistance technology purposes, based on a three-stage segmentation and characterization algorithm used to classify vascular, petechiae, trophic changes, and trauma macules from digital photographs of the lower limbs. First, in order to find the skin region, a logical multiplication is performed on two skin masks obtained from color space transformations. Then, in order to locate the lesion region, illumination enhancement is performed using a chromatic model color space, followed by a principal component analysis grayscale transformation. Finally, characteristics of each type of macule are considered and classified; morphologic properties (area, axes, perimeter, and solidity), intensity properties, and a set of shade indices (red, green, blue, and brown) are proposed as a measure to obviate skin color differences among subjects. E. Pociask et al. present a method aimed at the automated lumen segmentation on optical coherence tomography images. The adopted approach is composed of a preprocessing phase (artifacts removal: speckle noise, circular rings, and guide wire) and morphological operations. The method is tested on 667 images of different patients from the Medical University of Silesia, and it has been compared with other ones by using objective measures. R. Zhang et al. propose an improved fuzzy connectedness (FC) method for three-dimensional (3D) liver vessel segmentation on computed tomography (CT) images. In particular, a novel method to define the fuzzy affinity function of FC is presented, and an improved filter based on adaptive sigmoid filtering is proposed. The proposed approach is evaluated in 40 cases of clinical CT volumetric images from public image repositories. Y. Gao et al. proposed a novel technique to perform the optic disc detection on retinal images. The elaboration pipeline involves an initial rough segmentation based on saliency detection and largest object selection to define the optic disc initial contour. Subsequently, a method based on a deformable model refines the result. The effectiveness of such an approach has been evaluated on a public retinal image repository. A. Cruz-Bernal et al. propose a method for the automated detection of malignant calcifications on mammography images. The approach is based on the analysis of the cluster prominence (cp) feature histogram. Indeed, the calcifications on the mammography are characterized by high occurrences in the histogram. Therefore, the Vandermonde interpolation is used to obtain a function which models the cp histogram, and a KNN classifier finalizes the method. Z. Kong et al. propose a method to segment MR brain images by means of a convolutional neural network. Initially, a wavelet technique is used to extract the contours of different tissues such as skull, cerebrospinal fluid (CSF), grey matter (GM), and white matter (WM). Therefore, a convolutional neural network refines the segmentation results. B. Khagi and G.-R. Kwon proposed a method to segment MR brain images using SegNet, a convolutional neural network. Such a CNN is trained by using presegmented MR brain images of the OASIS free dataset. Z. Z. Wang et al. described a simple, yet effective approach to segment three-dimensional livers in computed tomography imaging. This research field is of particular interest because the described task requires considerable experience by the clinician and it is subject to personal interpretation. The proposed method is based on multiple thresholds through slope difference distribution, on Gibbs energy minimization to reduce inhomogeneity and on proper mathematical morphology operations to refine the segmented components, represented by their spline contours. This heuristic was fine tuned experimentally on a variety of real cases on public datasets. C.-Y. Lee et al. introduced a neural network approach to deal with sonograms in the case of breast cancer. Potential applications to reduce the amount of noise and intensity inhomogeneity are quite evident in this particular field. The proposed technique has been validated on two real datasets and comparing different network models. The images are passed to a stack denoised autoencoder-based classifier to enhance the contrast of the ultrasound signal. The extraction of the most promising features and the comparison with manually labeled data allowed to train the system, thus gaining a final accuracy equal to 85%. J. Hai et al. have addressed the problem of automatic breast cancer segmentation through fully convolutional networks, able to detect abstract features from the input data. Moreover, a nonstandard multiresolution approach led to definition of a general technique, independent of the tumor size. Indeed, while the usual MRA analysis is efficient for compression purposes, the redundant methodology chosen by the authors retains the original resolution of the mammograms, and therefore, it is particularly suited for their automatic segmentation. Experimental results are presented on a vast proprietary dataset of real clinical cases. E. Dandıl proposed a technique for the automatic detection and classification of lung cancer in computed tomography. A preliminary phase identifies candidate pulmonary nodules, which are segmented by self-organizing maps, investigated by principal component analysis and classified due to a probabilistic neural network. The computer-aided diagnostic system achieves high accuracy, sensitivity, and specificity on a publicly available dataset. W. Tan et al. have defined an interesting technique for the identification of pulmonary vascular structures in chest computed tomography. They proved that the region growing with maximum between-class variance segmentation is enough to select the regions of interest. The vascular components are therefore fully located through the fast marching method. Experimental comparisons were done against other well-known methods, and the accuracy of this technique is about 90%.

